# Associations of overweight, obesity and osteoporosis with ankle fractures

**DOI:** 10.1186/s12891-021-04607-9

**Published:** 2021-08-23

**Authors:** Anja M. Hjelle, Ellen M. Apalset, Jan-Erik Gjertsen, Roy M. Nilsen, Anja Lober, Grethe S. Tell, Pawel F. Mielnik

**Affiliations:** 1 Department of Rheumatology, Division of Medicine, District General Hospital of Førde, Førde, Norway; 2 Department of Global Public Health and Primary Care, University of Bergen, Bergen, Norway; 3 Bergen group of Epidemiology and Biomarkers in Rheumatic Disease (BeABird), Department of Rheumatology, Haukeland University Hospital, Bergen, Norway; 4 Department of Orthopedic Surgery, Haukeland University Hospital, Bergen, Norway; 5 Department of Clinical Medicine, University of Bergen, Bergen, Norway; 6 Faculty of Health and Social Sciences, Western Norway University of Applied Sciences, Bergen, Norway; 7 Department of Radiology, District General Hospital of Førde, Førde, Norway

**Keywords:** Ankle fracture, Danis-weber classification, Osteoporosis, Overweight

## Abstract

**Background:**

Studies exploring risk factors for ankle fractures in adults are scarce, and with diverging conclusions. This study aims to investigate whether overweight, obesity and osteoporosis may be identified as risk factors for ankle fractures and ankle fracture subgroups according to the Danis-Weber (D-W) classification.

**Methods:**

108 patients ≥40 years with fracture of the lateral malleolus were included. Controls were 199 persons without a previous fracture history. Bone mineral density of the hips and spine was measured by dual-energy x-ray absorptiometry, and history of previous fracture, comorbidities, medication, physical activity, smoking habits, body mass index and nutritional factors were registered.

**Results:**

Higher body mass index with increments of 5 gave an adjusted odds ratio (OR) of 1.30 (95% confidence interval (CI) 1.03–1.64) for ankle fracture, and an adjusted OR of 1.96 (CI 0.99–4.41) for sustaining a D-W type B or C fracture compared to type A. Compared to patients with normal bone mineral density, the odds of ankle fracture in patients with osteoporosis was 1.53, but the 95% CI was wide (0.79–2.98). Patients with osteoporosis had reduced odds of sustaining a D-W fracture type B or C compared to type A (OR 0.18, CI 0.03–0.83).

**Conclusions:**

Overweight increased the odds of ankle fractures and the odds of sustaining an ankle fracture with possible syndesmosis disruption and instability (D-W fracture type B or C) compared to the stable and more distal fibula fracture (D-W type A). Osteoporosis did not significantly increase the odds of ankle fractures, thus suffering an ankle fracture does not automatically warrant further osteoporosis assessment.

## Background

Ankle fractures constitute approximately every tenth fracture in adults [1, 2], and two thirds are a result of a low-energy trauma (equivalent to a fall from standing height or lower) [1]. A Swedish study of patients hospitalized due to ankle fractures from 1987 through 2004, found an average annual incidence rate of 71 per 100,000 person-years, and increasing fracture incidence over time in elderly women [3]. Ankle fractures are not considered to be typical osteoporotic fractures, although results from some studies do suggest otherwise [4, 5]. Compared to patients with typical osteoporotic fractures (distal radius, hip and spine), patients sustaining an ankle fracture are usually younger [6] and have a higher body mass index (BMI) [7]. Several studies have concluded that there is no association between ankle fractures and low bone mineral density (BMD) [8–12], while others have reported a lower BMD in ankle fracture patients compared to controls as well as alterations in bone quality [13, 14].

It is a mechanically plausible presumption that osteoporosis could predispose to specific types of ankle fractures. King et al. [15], concluded that osteoporosis or osteopenia was not significantly associated with a greater risk of a more proximal distal fibula fracture (D-W types B and C), which can be instable because of possible syndesmosis disruption, and in most cases such fractures require surgical intervention.

Large epidemiological studies have reported that high BMI is positively correlated with increased BMD and reduced risk of fragility fractures in both men and women [16, 17]. The generally accepted explanation for this is that a larger body weight induces greater mechanical loading on bone, with a consequent increase in BMD to accommodate the greater load [18]. However, when the mechanical loading effect caused by total bodyweight is removed, both fat mass and fat percentage are negatively correlated with bone mass [19–21], and obesity is no longer considered protective against fracture [22]. Fracture algorithms, such as FRAX®, may underestimate fracture probability in individuals with obesity because of their high BMI and subsequently higher relative BMD compared to the reference population [23]. Knowing that at least 50% of fractures occur in people with normal BMD or osteopenia [24], it is also important to focus on BMD-independent clinical risk factors in order to optimize fracture prevention. Especially fractures at bone sites with a large proportion of cortical bone, such as the ankle, are positively associated with obesity [25]. A plausible biomechanical explanation is that increased weight generates greater force during a fall, twist, or turn. The same forces may also contribute to increased risk of a more complex injury.

Data have been conflicting regarding the role of sex. Some studies report that men have increased risk of ankle fracture compared to women [26, 27], while more recent studies show a higher incidence among women [1, 3, 28, 29]. Smoking, alcohol consumption, degree of physical activity and polypharmacy are other patient related risk factors for ankle fractures which have been investigated, with variable conclusions [7–9, 13, 27, 30–33].

In this study we compared patients with acute ankle fracture to controls without previous fractures, aiming to investigate if overweight and/or osteoporosis increased the odds of ankle fractures, and in particular of instable distal fibula fracture, in adults.

## Methods

### Subjects

From March 1, 2012 until January 13, 2017, 108 consecutive patients over the age of 40 living in Sogn og Fjordane County with acute ankle fracture and treated at the Department of Orthopedic Surgery at Helse Førde Hospital Trust were included in a case control study. The study was primarily designed to explore the prevalence of celiac disease and positive transglutaminase 2 in patients with peripheral fractures compared to community-based controls never having sustained a fracture. Fracture patients willing to participate were referred to the Department of Rheumatology. For the controls, we were provided with lists of randomly selected individuals from Sogn & Fjordane county in the following age cathegories: 40–49 years, 50–59 years, 60–69 years, 70–79 years and 80 years or older by the Norwegian Population Registry. In case of any fracture in their past medical history (except fingers or toes), they were not included as control subjects in the study. The original case control study has been described in detail previously [34]. The participation rate was 40.9% among ankle fracture patients and 42.6% in the control group. For the control group, the only exclusion criterion was any previous fractures (except fingers and toes). The included ankle fractures were uni- or bimalleolar, but all had to involve the lateral malleolus. Trimalleolar ankle fractures were not included because of an assumed likelihood of difference in trauma mechanism. We included both patients with low energy fractures (equivalent to fall from standing height or lower) and fractures due to traumas with higher energy. All participants signed a written informed consent form, and the study protocol was approved by the Regional Committee for Medical and Health Research Ethics (REC West).

### Procedures and measurements

**Fig. 1:**
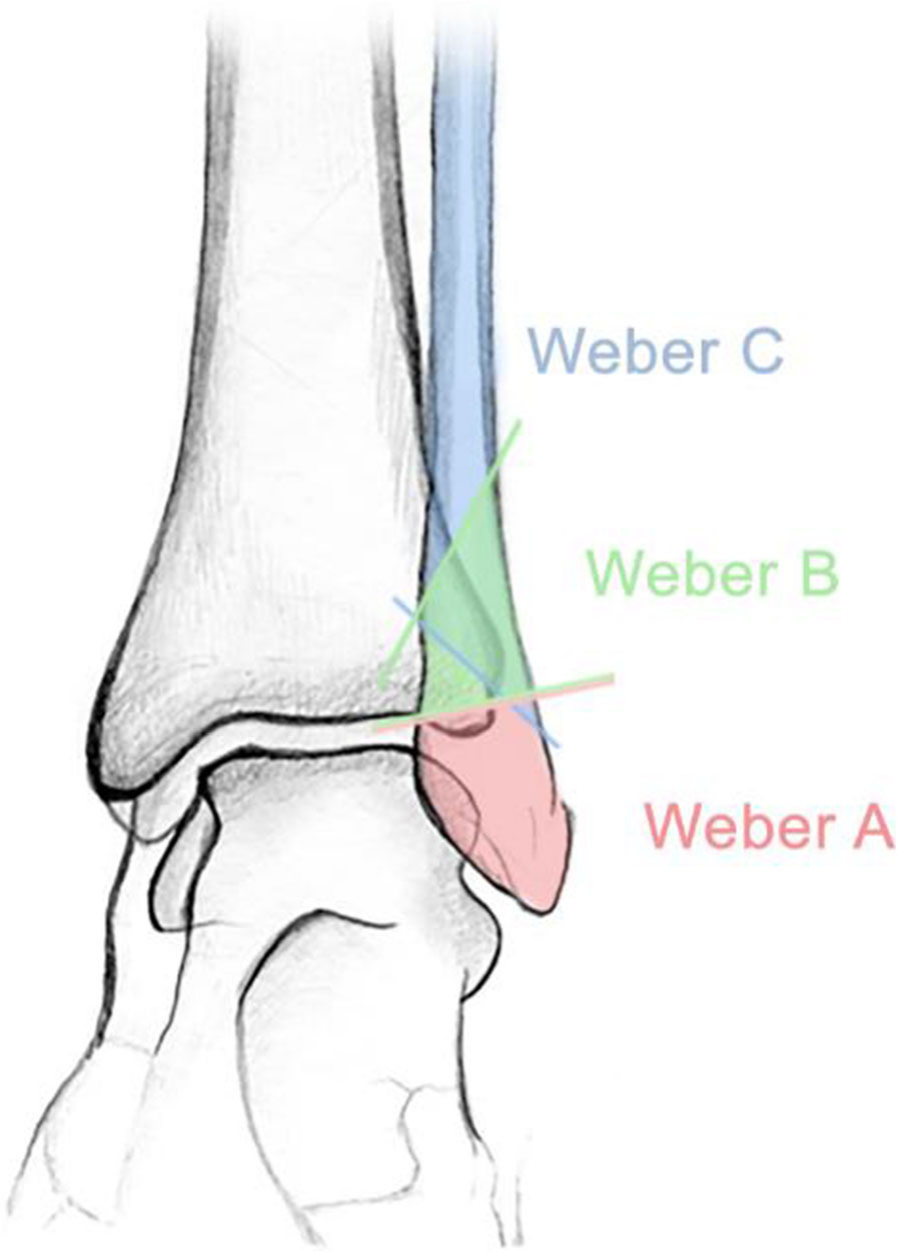
Illustration of the Danis-Weber classification of lateral malleolus fractures. Graphic design by Eir Pétursdóttir. *Type A: fracture of the lateral malleolus distal to the syndesmosis (usually stable). Type B: fracture of the fibula at the level of the syndesmosis (variable stability)*. *Type C: fracture of the fibula proximal to the syndesmosis (unstable)*

The BMD measurements were performed by DXA technology (Lunar Prodigy Rtg 5603, manufacture year 2000, GE Healthcare), with a daily quality assurance of +/− 2%. BMD was reported as g/cm^2^ and T-scores by standard definition [35]. Osteoporosis is defined as T-score ≤ − 2.5 in the femoral neck, total hip or lumbar spine. Osteopenia (low bone mineral density) is defined as T-score between − 1.0 and − 2.5 [36]. The radiographic ankle series comprised an anteroposterior, mortise (with the foot in 10 degrees internal rotation), and lateral radiographs. One experienced radiologist (AL) classified the ankle fractures as type A, B or C according to the Danis-Weber classification (D-W) (Fig. 1). D-W type A fractures occur below the level of the tibiofibular syndesmosis, leaving the syndesmosis and deltoid ligament intact. Type B fractures occur at the level of the syndesmosis, and may include injury of the syndesmosis, making the ankle joint unstable. Type C fractures occur above the level of the syndesmosis, result in disruption of the syndesmosis, and are defined as unstable. History of previous fractures, comorbidities, medication, and lifestyle factors were registered. Physical activity was assessed using the short form of the International Physical Activity Questionnaire (IPAQ) [37], categorizing the level of physical activity into high, moderate or low. The original documents from the orthopedic surgeons and examining rheumatologist were reviewed to classify the injury as due to a low energy trauma or not. Height and weight were measured as part of the DXA procedure. BMI was calculated and categorized into underweight (BMI < 18.5), normal weight (BMI 18.5–24.99), overweight (BMI 25–29.99), and obesity (BMI ≥ 30), [38]. Blood tests were analyzed to detect common causes of secondary osteoporosis [34]. Information about the BMD measurement was given to the patient during a consultation with one of the two rheumatologists in charge of the study on the day of examination, and appropriate treatment was either initiated or recommended to the patient and the patient’s general practitioner.

### Statistical analyses

We performed descriptive statistics for age, sex, height, weight, BMI, osteoporosis, osteopenia, smoking, physical activity quantified by the IPAQ score categories (HIGH: physical activity level equate to approximately 1 h or more of activity level of at least moderate intensity, MODERATE: activity more than likely equivalent to half an hour of at least moderate intensity on most days, and LOW: not meeting the criteria for moderate or high levels of physical activity), low energy trauma mechanism of injury (yes/no), 25-(OH) vitamin D levels and polypharmacy (defined as using three or more prescribed medications on a daily basis). Data for fracture patients were compared with controls using chi square or Fisher’s exact test for categorical data and two-sample t-test or Mann-Whitney U test for continuous data, as appropriate. To assess factors associated with fracture and Danis-Weber category, we estimated odds ratios (ORs) with 95% confidence intervals (CIs) using unconditional logistic regression models. All *p* values were two-sided, and values < 0.05 were considered statistically significant. All calculations were performed using IBM® SPSS Statistics Version 24, 2016 and R version 3.6.2 [39].

## Results

### Ankle fractures compared to controls

#### Overweight and BMI

**Fig. 2:**
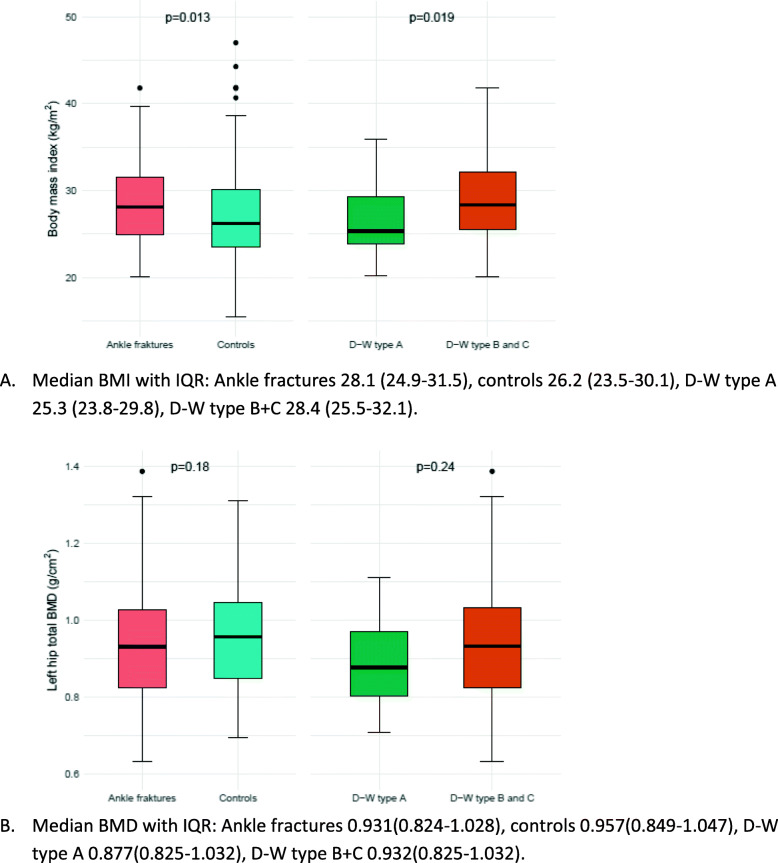
Box plot of BMI (**A**) and left hip BMD (**B**) in ankle fractures, controls and Danis-Weber subgroups. A Median BMI with IQR: Ankle fractures 28.1 (24.9–31.5), controls 26.2 (23.5–30.1), D-W type A 25.3 (23.8–29.8), D-W type B + C 28.4 (25.5–32.1). B Median BMD with IQR: Ankle fractures 0.931(0.824–1.028), controls 0.957(0.849–1.047), D-W type A 0.877(0.825–1.032), D-W type B + C 0.932(0.825–1.032). Centre horizontal line of the boxes represents the median. The boxes contain Q1 (25th Percentile) to Q3 (75th Percentile). IQR (Interquartile range) is the distance between Q1 and Q3. The bottom whiskers: less than Q1–1.5*IQR. The upper whiskers: greater than Q3 + 1.5*IQR

In the ankle fracture group, 27.1% had a normal body weight, 39.3% had overweight, and 33.6% had obesity as compared to 38.8, 34.5 and 25.8% in the control group, respectively. The fracture patients had a higher mean body weight compared to controls (Table 1). Median BMI was 28.1, compared to 26.2 in the control group (*p* = 0.013) (Fig. 2). Higher body mass index with an increment of 5 units was a significant risk factor for sustaining an ankle fracture (OR adjusted for age and sex 1.30 (95% CI 1.03–1.64)).

**Table 1:** Ankle fractures compared to controls

Characteristics	Ankle fractures compared to controls		
	Cases (*n* = 108)	Controls (*n* = 199)	*P*-value
Age			
Age, mean (SD^a^)	57.1 (9.9)	60.4 (10.5)	0.02
≥ 65, n (%)	25 (23.1)	69 (34.7)	0.06
Sex			
Male, n (%)	33 (30.8)	34 (17.3)	0.01
Female, n (%)	75 (69.2)	165 (82.7)	
Bone Mineral Density^b^ (DXA^c^)			
Osteoporosis^d^, n (%)	24 (22.4)	44 (22.3)	0.68
Osteopenia^e^, n (%)	52 (47.7)	103 (51.7)	
Normal BMD^f^, n (%)	32 (29.9)	52 (26.0)	
Height, weight, BMI^g^			
Weight, kg, mean (SD)	81.9 (17.2)	75.7 (15.1)	0.00
Height, cm, mean (SD)	168.8 (8.3)	166.6 (8.9)	0.04
BMI ≥ 25, n (%)	79 (73.2)	119 (60.1)	0.05
Smoking			
Smoking, current n (%)	21 (18.7)	22 (11.2)	0.11
Smoking, previous, n (%)	39 (36.4)	81 (40.6)	
Smoking, never, n (%)	48 (44.9)	96 (48.2)	
IPAQ-score^h^			
High^i^, n (%)	16 (14.8)	18 (9.0)	0.18
Moderate^j^, n (%)	69 (63.9)	148 (74.4)	
Low^k^, n (%)	23 (21.3)	37 (18.6)	
Low energy trauma^l^, n (%)	77 (71.3)		
25-(OH) Vitamin D^m^, mean (SD)	69.3 (23.0)	68.3 (21.6)	0.67
Polypharmacy (> 3)^n^, n (%)	25 (23.1)	43 (21.6)	0.80

^a^Standard deviation, ^b^Bone Mineral Density, ^c^Dual energy X-ray absorptiometry of the hips and spine, lowest measured T-score used, ^d^ T-score ≤ −2,5 ^e^T-score − 1.0 - -2.5, ^f^T-score ≥ −1.0, ^g^Body mass index, kg/m^2^, ^13^Defined as daily use of ≥3 prescribed medications, ^h^The International Physical Activity Questionnaire, ^i^physical activity equal to approximately one hour of moderate intensity activity per day or more, ^j^physical activity equal to approximately half an hour of moderate intensity activity on most days, ^k^physical activity lower than moderate IPAQ-score, ^l^equivalent to fall from standing height or lower, ^m^25-(OH) vitamin D as measured in nmol/L, ^n^daily use of three or more prescribed medications

*P*-values by chi square or Fisher’s exact test for categorical data and two-sample t-test or Mann-Whitney U test for continuous data, as appropriate

#### Bone mineral density

The prevalence of osteoporosis and osteopenia were similar in the ankle fracture group and the controls (22.4% vs 22.3 and 47.7% vs 51.7%, respectively) (Table 1). Median left hip total BMD was 0.931 g/cm^2^ in the subjects with ankle fracture, compared to 0.957 g/cm^2^ in the controls (Fig. 2A). Osteoporosis increased the odds of ankle fracture (adjusted OR 1.53 (95% CI 0.79–2.98), but the confidence interval was large (Table 3). We also compared the ankle fracture patients with osteoporosis to the fracture patients with normal BMD or osteopenia. Patients with ankle fracture and osteoporosis had lower BMI compared to patients with normal BMD/osteopenia (BMI 25.8 (SD 4.03) vs. 29.5 (SD 4.94), *p* < 0.001)), whereas there were no significant differences in gender, smoking habits or high vs. low energy trauma mechanism. In the ankle fracture patients with osteoporosis, 76.0% of the fractures were the result of a low energy trauma, compared to 69.9% in the non-osteoporotic patients (*p* = 0.63).

#### Other characteristics

The ankle fractures were in 71.3% of the cases caused by a low energy trauma (Table 1). The patients with ankle fractures were somewhat younger than the controls, and a higher percentage were men (*p =* 0.01). The 25- (OH) vitamin D levels were similar in the ankle fracture group and the control group, and the mean level were adequate. The proportions using three or more daily prescribed medications and the level of physical activity as assessed by the IPAQ were also similar between the two groups. The prevalence of current smokers in the ankle fracture group was somewhat higher compared to the controls (Table 1).

### Danis- weber type B and C compared to type a

#### Overweight and BMI

When examining the patients with ankle fractures D-W type A, 47.0% had normal body weight, 35.3% were overweight, and 17.7% had obesity. The corresponding numbers for D-W type B were 25.4, 36.6 and 38.0%, and for D-W type C 15.0, 45.0 and 40.0%, respectively. The patients with D-W type B and C had a higher mean body weight compared to the group with D-W type A, and overweight was more prevalent (*p* = 0.02) (Table 2). Median BMI in the D-W type A group was 25.3, compared to 28.4 in the D-W type B and C group (*p* = 0.02) (Fig. 2). Higher body mass index with an increment of 5 units associated with increased odds for ankle fracture (crude OR 2.11 (95% CI 1.15–4.33), OR adjusted for age and sex 1.96 (CI 0.99–4.41)).

**Table 2:** Ankle fracture Danis-Weber Type A compared to Type B or C

Characteristics	Danis-Weber classification (D-W)		
	D-W A (*n* = 17)	D-W B or C (*n* = 91)	*P*-value
Age			
Age, mean (SD^1^)	57.0 (9.1)	57.5 (10.2)	
≥ 65, n (%)	8 (47.1)	70 (76.9)	0.79
Sex			
Male, n (%)	7 (41.2)	26 (28.6)	0.45
Female, n (%)	10 (58.8)	65 (71.4)	
Bone Mineral Density^b^ (DXA^c^)			
Osteoporosis^d^, n (%)	8 (47.1)	17 (18.7)	0.03
Osteopenia^e^, n (%)	6 (35.3)	35 (38.5)	
Normal BMD^f^, n (%)	3 (17.6)	39 (42.8)	
Height, weight, BMI^g^			
Weight, kg, mean (SD)	75.7 (14.1)	83.1 (17.5)	0.13
Height, cm, mean (SD)	170.0 (7.6)	168.5 (8.5)	0.42
BMI ≥ 25, n (%)	9 (52.9)	70 (76.9)	0.02
Smoking			
Smoking, current n (%)	4 (23.5)	17 (18.7)	0.65
Smoking, previous, n (%)	7 (41.2)	32 (35.2)	
Smoking, never, n (%)	6 (35.3)	42 (46.1)	
IPAQ-score^h^			
High^i^, n (%)	4 (23.5)	12 (13.2)	0.39
Moderate^j^, n (%)	9 (52.9)	60 (66.0)	
Low^k^, n (%)	4 (23.5)	19 (20.1)	
Low energy trauma^l^, n (%)	12 (70.6)	65 (71.4)	1.0
25-(OH) vitamin D^m^, mean (SD)	69.7 (22.2)	69.2 (23.3)	0.56
Polypharmacy (≥3)^n^, n (%)	3 (17.6)	22 (24.2)	0.76

^a^Standard deviation, ^b^Bone Mineral Density, ^c^Dual energy X-ray absorptiometry of the hips and spine, lowest measured T-score used, ^d^ T-score ≤ −2,5 ^e^T-score − 1.0 - -2.5, ^f^T-score ≥ −1.0, ^g^Body mass index, kg/m^2^, ^13^Defined as daily use of ≥3 prescribed medications, ^h^The International Physical Activity Questionnaire, ^i^physical activity equal to approximately one hour of moderate intensity activity per day or more, ^j^physical activity equal to approximately half an hour of moderate intensity activity on most days, ^k^physical activity lower than moderate IPAQ-score, ^l^equivalent to fall from standing height or lower, ^m^25-(OH) vitamin D as measured in nmol/L, ^n^daily use of three or more prescribed medications

*P*-values by chi square or Fisher’s exact test for categorical data and two-sample t-test or Mann-Whitney U test for continuous data, as appropriate

#### Bone mineral density

There was a higher prevalence of osteoporosis in the D-W A fracture group (47.1%) compared to the subjects with D-W type B and C (18.7%) (*p* = 0.03) (Table 2). Median left hip total BMD was 0.877 g/cm^2^ in the D-W type A cases, compared to 0.932 g/cm^2^ in D-W type B and C subjects (*p* = 0.24) (Fig. 2). Ankle fracture patients with osteoporosis had reduced odds of sustaining a D-W fracture type B or C compared to type A (OR 0.18, (0.03–0.83). A similar tendency, although less pronounced, was seen in the case of osteopenia (adjusted OR 0.39, (CI 0.07–1.71) (Table 3).

**Table 3:** Odds of ankle fractures compared to controls, and of Danis- Weber types B and C compared to type A

Characteristics	Ankle fractures (*n* = 108) compared to controls (*n* = 199)		Danis-Weber B and C (*n* = 91) compared to Danis-Weber A (*n* = 17)	
	Crude OR (95% CI)	Adjusted^a^ OR (95% CI)	Crude OR (95% CI)	Adjusted^1^ OR (95% CI)
BMI^b^, per 5 unit change	1.29 (1.03–1.65)	1.30 (1.03–1.64)	2.11 (1.15–4.33)	1.96 (0.99–4.41)
Osteoporosis^c^	1.18 (0.64–2.16)	1.53 (0.79–2.98)	0.16 (0.03–0.64)	0.18 (0.03–0.83)
Osteopenia^d^	1.26 (0.74–2.15)	1.29 (0.74–2.27)	0.45 (0.09–1.83)	0.39 (0.07–1.71)

^a^Adjusted for age and sex, ^b^Body mass index, kg/m^2^, ^c^T-score ≤ − 2,5 ^d^T-score − 1.0 - -2.5

ORs are estimated using multivariable logistic regression models. Whiskers represent 95% confidence intervals

Reference category for osteoporosis was no osteoporosis (osteopenia and normal bone mineral density). Reference category for osteopenia was normal bone mineral density (T-score ≥ − 1.0)

#### Other characteristics

Comparing D-W type A fractures with D-W type B and C fractures, there was no difference in the proportion of fractures caused by a low energy trauma. The mean age, sex distribution, smoking status, physical activity level, 25-(OH) vitamin D and the proportion of patients using three or more daily prescribed medications were similar between the two groups (Table 2).

## Discussion

In this study, we found that people with overweight or obesity had increased odds of ankle fractures, compared with normal weight people. The role of osteoporosis in ankle fractures is less clear. Overweight increased the odds of sustaining an ankle fracture with possible syndesmosis disruption and instability (D-W fracture type B or C) compared to the stable and more distal fibula fracture (D-W type A), while osteoporosis seemed to have a protective effect on D-W type B and C.

Obese individuals have an increased risk of falls. Intramuscular fat content is associated with poorer muscle function and postural instability [40]. There may also be impairment in the normal protective responses during a fall, and a predisposition to fall sideways or backwards [41]. Increased weight generates a greater force during a fall or another sudden body movement, and subsequently, a greater probability of ankle fracture. And, although BMD measured by DXA is higher in obesity, it may not be sufficiently high to resist the greater forces acting during the fall [40]. Furthermore, it may be speculated that other factors promoting a falling tendency, such as the use of alcohol or physical inactivity, may accentuate the BMI effect. Lacombe et al., however, did not find evidence for interactions between BMI and physical activity for fracture risk [42]. How fat distribution and obesity impact bone health and fracture risk is complex, and not fully understood.

Our study could not demonstrate a clear correlation between osteoporosis or osteopenia and the odds of sustaining an ankle fracture. None of the classical risk factors of fractures strongly associated with osteoporosis, such as increasing age, female sex, vitamin D deficiency, and low BMI, could be identified as risk factors for ankle fracture. This supports previous studies concluding that ankle fractures cannot be considered classical osteoporotic fractures. Roux et al. [43] found that personal history of osteoporosis was less frequent (*p* < 0.001) for ankle fragility fractures vs fragility fractures of the wrist and at other sites.

We found that men had increased odds of ankle fracture compared to women, and individuals under the age of 65 seem to be at greater risk of sustaining an ankle fracture compared to those ≥65 years of age. Our results are in line with Roux et al. [43], who found that patients with ankle fracture were significantly younger, more likely to be male, and had higher BMI. Liu et al. [30] found that alcohol consumption, living alone and sleep time < 7 h a day were risk factors for ankle fracture, and these factors did not differ between genders. We could speculate that a higher alcohol consumption, especially in younger age groups, as well as more outdoor activities, both sports [27, 31] and work related, are factors that might contribute to a higher ankle fracture incidence in men. Smoking is also related to other behavioral factors (like alcohol and drug use) that may increase the risk of injury. In our study, smoking was not significantly associated with ankle fractures. Valtola et al. looked at ankle fractures in perimenopausal women (mean age 52.3), and found the use of ≥3 prescribed drugs to be an independent predictor for malleolar fracture (in addition to smoking, overweight and previous fracture) [7]. In our study, the proportion of individuals with daily use of ≥3 prescribed drugs did not differ between the ankle fracture patients and the controls.

We found no association between the level of physical activity, as assessed by the IPAQ, and risk of ankle fractures. However, the relationship between physical activity and risk of fracture is complex and multifaceted, especially when considering the individual fracture sites. Physical activity may protect against falls through improved balance, muscle strength and coordination. On the other hand, participating in regular physical activity, people are at an increased risk of falls that may lead to injury [42].

### Danis- weber types B and C compared to type a

To our knowledge, the only previous study addressing the association between BMD measured by DXA and the subgroups of ankle fractures according to the D-W classification is by King and colleagues [15]. They investigated 280 patients ≥25 years with ankle fracture, and concluded that osteoporosis/ osteopenia was not associated with increased complexity of the lateral malleolar fracture, which is in concordance with our results. In fact, we found that osteoporosis resulted in a higher odds for Type A compared to Type B and C, patients with osteoporosis had fewer instable lateral malleolar fractures than those with osteopenia and normal BMD. We are not aware of other clinical or biomechanical studies regarding ankle fractures describing similar results. This is an interesting finding. We might speculate, that patients with osteoporosis, having reduced cortical thickness in the metaphyseal area, could be more prone to fracture closer to the metaphysis as opposed to more proximally.

Patients with overweight in our study had greater odds of having a type B or C fracture compared with type A, as the study by King et al. also showed. This is mechanically plausible, since high body weight adds to the force in a fall or an ankle sprain, and therefore may lead to more severe injury.

### Strengths and limitations

The controls were from the same geographical area as the patients, considered important since previous studies from Norway have shown significant regional differences in hip BMD [44]. The use of population based controls instead of hospital-based, limits selection bias. All DXA measurements were performed on the same machine by the same experienced nurse in our osteoporosis clinic, which decreases the risk of measurement variance. The controls and the fracture patients were analyzed in the same time period, minimizing the risk of measurement drift and changes in laboratory methods. All patients and controls had a consultation with one of two experienced rheumatologists on the same day as the DXA analysis, where the extensive questionnaire was reviewed and lacking information could be supplemented. Thus, the information on known and potential confounding factors is extensive.

The main study was not primarily designed to investigate the aims of the current study, and power analysis were not performed for these research questions. The results primarily apply for our group of fracture patients and controls, and precautions in generalizability should be taken. Some of the patients and controls who chose not to participate in the study may already have been diagnosed with osteoporosis, and therefore did not consider the participation as meaningful. If this differed between the fracture patients and the control group, this may have been a source of selection bias. The prevalence of osteoporosis in the study might, for the same reason, be underestimated in both groups. However, this might affect the inclusion rate of both fracture patients and controls. Another possible selection bias is that fracture patients were asked to participate face to face by a treating physician, whereas the controls were invited by letter only. As in all case-control study designs, recall bias is a concern in the collection of retrospective data, for example life style factors and self-reported comorbidities.

We chose to combine the D-W B and C subgroups, these fracture subtypes being at risk of instability because of syndesmosis disruption. However, these two subgroups may differ in both the trauma mechanism responsible for the injury, and in patient characteristics. There are few cases in the D-W type A fracture group, and the study may consequently be underpowered regarding fracture subgroup comparison. We did not have a sufficient number of fracture patients in order to investigate if the use of specific groups of prescribed medication were associated with increased fracture risk.

## Conclusions

Having a BMI over 25 increased the odds of ankle fractures in this case control study, while we cannot conclude with a similar association with osteoporosis and ankle fractures. Overweight also increased the odds of a D-W type B and C fracture compared to the stable and less severe type A fracture of the distal fibula. Patients with osteoporosis, however, had reduced odds of sustaining a D-W fracture type B or C compared to type A. The association between overweight and ankle fracture can most likely be explained by biomechanical factors, but other factors increasing the fracture risk in this population are also important to consider in a fracture preventing approach. According to our results, ankle fractures cannot be considered a classical osteoporotic index fracture, and the occurrence of an ankle fracture alone does not indicate a need for further osteoporosis assessment.

## Data Availability

Due to regulations from the Norwegian Data Inspectorate and according to Norwegian personal protection laws, publication of the complete dataset is not legal or appropriate. If authors/researchers wish to have access to the dataset, this can still be achieved through direct contact with us.
